# Assessing patient partnership among emergency departments in France: a cross-sectional study

**DOI:** 10.1186/s12913-023-09905-7

**Published:** 2023-08-23

**Authors:** Geoffrey Sagnol, Julie Haesebaert, Anne Termoz, Philipe Michel, Anne-Marie Schott, Véronique Potinet, Marie-Pascale Pomey, Karim Tazarourte, Marion Douplat

**Affiliations:** 1 Hospices Civils de Lyon, Service d’Accueil des urgences, Hôpital Lyon Sud, 165 chemin du Grand Revoyet, Pierre Bénite, F-69495 France; 2 Pôle de Santé Publique, service de recherche et d’épidémiologie cliniques, Hospices Civils de Lyon, Lyon, France; 3 University Claude Bernard Lyon , U1290 Reshape, Lyon (Rhône), France; 4 Hospices civils de Lyon, Lyon, 69002 France; 5 Department of Health Policy, Management and Evaluation, School of Public Health, University of Montreal, Montreal, QC Canada; 6 Hospices Civils de Lyon, Service d’Accueil des urgences, Hôpital Edouard Herriot, 5 place d’Arsonval, Lyon, F-69003 France; 7 UMR 7268 ADéS, Aix-Marseille Université / EFS / CNRS, Espace éthique méditerranéen, Hôpital Adultes La Timone, 264 rue Saint Pierre, Marseille cedex 05, France

**Keywords:** Emergency departments, Partnership, Cross-sectional studies

## Abstract

**Objectives:**

This study aims to describe the use of patient partnership, as defined by the Montreal Model, in emergency departments (EDs) in France and report the perception of patient partnership from both the practitioner and patient perspectives.

**Methods:**

This cross-sectional study was conducted between July 2020 and October 2020. First, a survey was sent to 146 heads of EDs in both teaching hospitals and non-teaching hospitals in France to assess the current practices in terms of patient partnership in service organization, research, and teaching. The perceived barriers and facilitators of the implementation of such an approach were also recorded. Then, semi-structured telephone interviews were carried out with patients involved in a service re-organization project.

**Results:**

A total of 48 answers (response rate 32.9%) to the survey were received; 33.3% of respondents involved patients in projects relating to service re-organization, 20.8% involved patients in teaching projects, and 4.2% in research projects. Overall, 60.4% of the respondents were willing to involve patients in re-organization or teaching projects. The main barriers mentioned for establishing patient partnership were difficulties regarding patient recruitment and lack of time. The main advantages mentioned were the improvement in patient/caregiver relationship and new ideas to improve healthcare. When interviewed, patients mentioned the desire to improve healthcare and the necessity to involve people with different profiles and backgrounds. A too important personal commitment was the most frequently raised barrier to their engagement. All the patients recognized their positive role, and more generally, the positive role of patient engagement in service re-organization.

**Conclusion:**

Although this preliminary study indicates a rather positive perception of patient partnership among heads of EDs in France and partner patients, this approach is still not widely applied in practice.

**Supplementary Information:**

The online version contains supplementary material available at 10.1186/s12913-023-09905-7.

## Introduction

Patient-centered care is a fundamental concept in healthcare, which states that practitioners and patients should work in partnership to ensure that the resulting decisions are compassionate, empathetic, and in line with the patients’ values and preferences [1]. This approach could lead to better health outcomes [2] and improve patient safety, satisfaction, and well-being [3]. Between the years 2010 and 2015, greater attention was placed on patient engagement [4] which led to the design of various interventions aiming to develop patient partnership [3, 5–8].

In order to better structure this practice, Carman et al. proposed a framework for developing interventions and policies that support patient and family engagement [4]. The Montreal Model was then developed based on the recognition that patient experiential knowledge is complementary to the scientific knowledge of healthcare professionals [9–11]. According to this model, the patient could be considered as a healthcare provider, an equally valued member and partner of the healthcare team [10]. The partnership between patients and healthcare professionals can then be viewed as a continuum of patient involvement that begins with information sharing and culminates in partnership [11]. The involvement of patients in healthcare can occur at multiple levels: macro (governance or health policies), meso or organizational (design of healthcare services), and micro or clinical (peer support) [9]. They can also be involved in other settings of the healthcare system such as professional training, education, and research. In France however, although the AIDS epidemic sped up patient engagement in the 1980s, the partnership as described in the Montreal Model is still not widely applied [12, 13].

The context of emergency departments (EDs) is singular and faces many challenges such as time management constraints and a chaotic work environment which can greatly impact patient care. In this context, sharing the decision-making process with patients is often difficult [14]. It is however, of vital importance that patients and families who use the EDs are involved in ED care as well as other health-related domains, such as research [14, 15].

Recently, the Lyon university hospital decided to involve patients working with healthcare teams in the co-design a new ED building. We thus wished to gather patients’ views on their involvement to illustrate patient partnership at the service organizational level as defined by the Montreal model.

This exploratory study aimed to provide cross-sectional data on patient partnership practice in French EDs, both from the practitioner and patient perspectives. The aim of this study was twofold: provide a description of current patient engagement in French EDs based on the Montreal Model regarding service organization, teaching, and research as well as describe the perception of patients who were involved in healthcare decisions at an organizational level.

## Methods

### Study design

This cross-sectional study was conducted to obtain data on patient partnership in France from the perspectives of both the practitioners and the patients. First, between July 2020 and October 2020, a survey was sent to heads of EDs in France in order to obtain data regarding the current implementation of patient partnership at three levels: service organization, teaching, and health research. Then, a semi-structured interview with patients participating in the design of a future building in a French teaching hospital was conducted to obtain their perspective regarding their involvement. The present report follows the STROBE statement for cross-sectional studies.

### Survey

An online survey was developed to assess the implementation of patient partnership among EDs. The questionnaire was designed by two ED physicians and a public health physician, driven by the patient engagement continuum of the Montreal Model and based on the available literature. From July to October 2020, the survey was sent to the professional email addresses of all the heads of EDs, in both teaching hospitals and non-teaching hospitals, of each county capital city in France, representing the largest French hospitals. Out of the 32 French teaching hospitals, the contact for 3 of them could not be obtained. Among the 488 non-teaching hospitals in France, 117 from the county capital cities were contacted.

The questionnaire (detailed in the supplemental material S1) is composed of 26 questions assessing the respondent’s practices and views regarding patient engagement in service organization, research, and training of health students, based on the Montreal Model. Six questions assessed their perspective on patient engagement. The point of view of the respondents regarding patient involvement in service organization, research, and training was measured using a Likert scale between 1 (strongly agree) and 5 (strongly disagree). To analyze the results, the 5 categories were grouped into 3 categories: “agree + strongly agree”, “disagree + strongly disagree”, and “undecided”. Respondents were also asked to give three advantages and three disadvantages, ranked by importance, for patient engagement as well as their willingness to apply patient partnership in their department.

### Semi-structured interviews

Qualitative data were collected though semi-structured interviews conducted via individual telephone calls in October 2020. The interviewees were patients participating in meetings for the design of a future building in the Lyon teaching hospital.

The interviewer was an ED physician who did not have a previous relationship with any of the participants. After receiving the participants’ agreement, all interviews were recorded using a microphone, made anonymous, and later transcribed verbatim by the interviewer. The interview guide was developed by two ED physicians, a public health physician, and a social psychologist based on the Montreal Model and available literature [6]. The interview guide was then reviewed by a partner patient who was not involved in the study. The interview guide is detailed in the supplemental material (S2).

### Statistical analysis

Data from the online survey were described using frequencies and proportions for categorical variables, and mean and standard deviations were used to describe quantitative variables. Results were described globally and by hospital type (teaching and non-teaching). Answers to open ended questions were divided into categories using inductive content analysis. An overall score was then attributed to each category by summing the points determined by the number of occurrences and the rank in the answer: 3 points for the first rank, 2 points for the second rank, 1 point for the third rank.

The qualitative analysis of the interviews was carried out by an ED physician and a social psychologist using MAXQDA2020^®^. The categories were previously defined using the interview grid and the study objectives. Subsequently, MAXQDA2020^®^ was used to distribute fragments of the interviews into different categories, thereby allowing sub-categories to be defined and thus bringing out key ideas.

## Results

### Online survey

Of the 146 EDs contacted, a total of 48 responses (32.9%) were received; 20/29 (69%) and 28/117 (23.9%) answers were obtained among the target population of the teaching and non-teaching hospitals, respectively. Among the medical heads of EDs, 17 (35.4%) were women and 28 (58.3%) worked in a non-teaching hospital (Table 1). Among the teaching and non-teaching hospitals, 12/20 (60%) and 6/28 (21.4%) heads of EDs knew the patient partnership approach, respectively.

**Table 1:** Characteristics of the heads of emergency departments who answered the survey

Characteristics		n = 48
Sex	Female	17 (35.4%)
Age	< 35	5 (10.4%)
	35–55	30 (62.5%)
	> 55	13 (27.1%)
Years in practice of emergency medicine	< 10	8 (16.6%)
	10–20	20 (41.7%)
	> 20	20 (41.7%)
Type of hospital	Teaching hospital	20 (41.7%)
	Non-teaching hospital	28 (58.3%)

Data are expressed as N (%)

### Patient involvement in service organization

Among all respondents, 16 (33.3%) reported involving patients in service organization. Patient engagement was performed mainly by surveys conducted on patients (13/16) and through consulting patient committees (6/16). The main type of projects involving patients related to developing care pathways (Table 2).

**Table 2:** Type of patient involvement in service organization reported by the heads of emergency departments

Patient involvement in service organization		n = 16
How patients were involved	Surveys conducted on patients	13
	Consulting patient committees	6
	Personal interviews or focus groups	4
	Working groups involving patients and caregivers	3
	Other	2
Type of project	Developing care pathways (protocols, documents for patients…)	14
	Service reorganization	9
	Installing signposting	6

The expected benefits of patient involvement in service organization are shown in Table 3.

**Table 3:** Expected benefits reported by the heads of emergency departments regarding patient involvement in service organization

Expected benefits of patient involvement in service organization		Strongly disagree/ disagree	Undecided	Agree/ strongly agree
Patient involvement could optimize patient pathway	Teaching hospitals (n = 20)	5 (25%)	4 (20%)	11 (55%)
	Non-teaching hospitals (n = 28)	2 (7%)	11 (40%)	15 (53%)
	Total (n = 48)	7 (15%)	15 (31%)	26 (54%)
Patient involvement could improve the functional and spatial organization of the premises	Teaching hospitals (n = 20)	9 (45%)	3 (15%)	8 (40%)
	Non-teaching hospitals (n = 28)	5 (18%)	9 (32%)	14 (50%)
	Total (n = 48)	14 (29%)	12 (25%)	22 (46%)
Patient involvement could improve patient experience regarding care	Teaching hospitals (n = 20)	4 (20%)	4 (20%)	12 (60%)
	Non-teaching hospitals (n = 28)	3 (11%)	6 (21%)	19 (68%)
	Total (n = 48)	7 (14%)	10 (21%)	31(65%)

Data are expressed as N (%)

### Patient involvement in research

Among all respondents, 35 (72.9%) had a research activity in their department and 4 (8.3%) reported involving patients in research. How patients were involved, at which stages, and the type of patients recruited are reported in Table 4. When respondents were asked if they believed that taking into account the patients’ perspective was important for the feasibility/implementation of research protocol, 6/20 (30%) in the teaching hospitals and 8/28 (29%) in the non-teaching hospitals agreed and strongly agreed.

**Table 4:** Type of patient involvement in research reported by the heads of emergency departments

Methods of patient involvement in research		n = 4
Patients already involved in feasibility or implementation of research protocols	Teaching hospitals	2/4
	Non-teaching hospital	2/4
How patients were involved	Surveys conducted on patients	2/4
	Personal interviews or focus groups	1/4
	Consulting patient committees	1/4
	Working groups involving patients and caregivers	1/4
Research stages at which patients are involved	Study pilot committee	1/4
	Protocol development	1/4
	Ethical considerations	1/4
	Patient enrollment	2/4
	Result reporting	1/4
Type of patients involved	Individual patients	3/4
	Patient representatives	2/4
	Patient relatives	1/4

### Patient involvement in training of healthcare students

Overall, 10 respondents (20.8%) involved patients in the training of health students, 7 of whom worked in teaching hospitals. Thirty-four respondents (70.3%) viewed the impact of patient involvement on medical students and residents as positive and 31 (64.5%) viewed this impact as positive on nursing and care assistant students. When asked if patient experience is formative for health students in EDs, 16/20 (80%) respondents from teaching hospitals and 16/28 (57%) from non-teaching hospitals agreed or strongly agreed.

The categories emerging from the perceived advantages sorted by the highest rank to the lowest were: caregiver/patient relationship (40), fresh perspective for caregivers (26), patient experience/comfort (24), observance, acceptance, and understanding of care (17), care quality (15), understanding and targeting the specific needs of the patients (14), improving service organization (12), redefining the place of patients in healthcare (8), education/training of health students (5), better understanding of the patient of healthcare system (5) and improving the influence of caregivers on the institutions (4).

The categories emerging from the perceived disadvantages sorted by the highest rank to the lowest were: difficulties in recruiting patients (26), lack of time (21), poor understanding of issues related to healthcare and healthcare system (19), representativeness of patients (11), ineffectiveness of the patient partnership approach (11), difficulties in setting up the organization (9), lack of methodological support (8), patient compensation and funds allocated (7), temporality and organization of EDs not in favor (6), medical secrecy (6), intervention of patients with a negative attitude (5), acceptance of care professionals (5), communication issues between the caregivers and the patients (4), diverging objectives among patients and caregivers (4).

The results regarding the willingness of respondents to integrate patients as partners are reported in Table 5.

**Table 5:** Respondents willing to involve patients

Willingness for patient involvement		n = 48
Service organization	Teaching hospitals	12 (60%)
	Non-teaching hospitals	17 (60.7%)
Research	Teaching hospitals	11 (55%)
	Non-teaching hospitals	5 (17.9%)
Teaching/training	Teaching hospitals	15 (75%)
	Non-teaching hospitals	14 (50%)

Data are expressed as N (%)

### Semi-structured interviews

A total of 12 patients involved in the service re-organization project were contacted, 6 of whom responded (50%). The 6 interviewees were all retired from work, none of them were involved in patient associations. Concerning their ED experience, all had interacted with the ED in different ways. Four had accompanied a relative to the ED, one had a personal experience in an ED, one visited several EDs through his work, and two were followed-up at the same hospital where the ED project was carried out.

When commenting on how the project workshops were carried out, all the interviewees mentioned that they valued the clarity of the explanations and the role of each participant. The convivial setting was highly appreciated. One participant reported feeling more comfortable participating in a small group.

*“I expressed my feelings, it was taken into account. We were a person. There was no feeling of being superior or inferior. Everyone played his role, everyone introduced himself and everyone talked about the project in their own way. Bravo. This is a good experience.“ (Interview 2)*.

50% of the interviewees highlighted that the exchanges with the meeting participants, including the architect, were valuable and interesting. *“I found it very interesting to see what was happening from the inside, and to understand why things were not done quickly. It’s easy to say but more difficult to act. (Interview 4)*

Two participants particularly appreciated the participation of different professions including doctors, technical staff, and the architect. *“All the trades were represented. Even the technical staff. If you have a question to ask, you raise your hand and you can participate.“ (Interview 2)*.

All the interviewees felt legitimate and able to participate. All but one felt competent enough to participate. All the interviewees but one felt their work valued, although they all recognized a modest, but meaningful contribution. “Between us, mute the sound, my contribution was decisive *laughing*. I assume that everyone’s participation, even the smallest, contributes to develop a project. Small streams make big rivers.“ (Interview 5).

All interviewees recognized a positive role of patient engagement in service organization.

One interviewee said they had no opinion regarding the involvement of patients in medical student training. Another interviewee said they did not feel competent enough to train medical students. The others welcomed the idea. *“This is a very technical field but the purpose is always the patients. So it’s a good thing to involve them, but not as potted plants. But the modalities need to be defined. At which level, the medical students from the beginning? The medical residents?…” (Interview 5)*.

Four out of the six interviewees considered patient involvement in research a good idea. One of them pointing out the necessity of recruiting “selected” patients.

*“Of course, when a study is conducted, I think that you need to consider every aspect, not just medical ones. Patient perspectives are interesting, his healthcare pathway, what went well, whether he suffered… My last operation I urinated blood. It was painful. I was expressing myself, I was scared. This aspect is very important too.“ (Interview 2)*.

Among the facilitators reported for patient engagement, the interviewees first mentioned the desire to improve healthcare. They then mentioned the need of diverse profiles with different perspectives in a healthcare system. Two participants wanted to share their experience. Two interviewees considered the meetings as a means to exchange ideas with different types of people. For two patients, personal curiosity was a driving engine. *“The aim is to be constructive, bring his little contribution and enjoy yourself. And meet people too.“ (Interview 5)*.

For the interviewees, the obstacles to patient engagement included too much personal involvement, a lack of competence, a lack of consideration for patient perspectives, and poor health.

All the interviewees were willing to continue working on the project. Two of the patients reported that they would agree to participate in a future project. One of the interviewees spontaneously volunteered to play a part in other projects.

## Discussion

This cross sectionnal study provides an overview of patient partnership practices in France and describes the perspective of partner patients in a specific project relating to service organization. The findings herein show that the patient partnership approach is not widespread among French EDs, and is mostly practiced at teaching hospitals. However, the results from the survey highlight a rather positive perception of patient engagement among heads of EDs, who view this approach as likely to improve patient experience. A majority of respondents were willing to integrate patients as partners in programs concerning service organization and training/education. The reported perceptions of patient engagement in research by the heads of EDs however, are less clear-cut. From the patient perspective, patient engagement in service organization was described as positive, the patients reporting feeling legitimate and competent in this role. However, the perception regarding patient engagement in medical student training was more mixed. Regarding involvement in research, patients reported their willingness to be involved.

### Survey

There are few data in the literature concerning the involvement of patients as partners in EDs and to our knowledge, the present study is the first to provide an overview of patient partnership practices in EDs in France. According to the findings herein, the current way of implementing patient engagement in service organization are mainly low-level engagements, corresponding to patient consultation in the patient engagement continuum defined by the Montreal Model [9]. Traditionally, the gathering of patient perspective was developed in EDs to assess patient satisfaction regarding quality of care [16, 17]. The literature however, describes a shift from patient satisfaction to patient experiences regarding quality of care [18]. Moreover, it has been shown that involving patients has an impact on quality of care and that the level of engagement influences the outcomes in terms of service re-organization; the higher the level of engagement the more care process or structural outcomes are observed [6]. A recent study evaluating patient engagement practices in a French teaching hospital showed that interventions involving patients were more often concerned with healthcare quality and safety rather than education and research, which is in line with the present findings [13].

Studies concerning patient engagement in research can help align research topics to match patient priorities, improve survey and data collection tools, increase patient recruitment and participation in studies, and improve dissemination of clinically relevant results from medical research [19]. The survey herein shows a very low prevalence of patient engagement in research, which is consistent with the international literature [20]. The barriers concerning patient involvement were mostly difficulties concerning patient recruitment and the lack of evaluation of patient engagement methods in research. The group of Wright et al. have proposed to adapt the framework for patient partnership in research [21] to the contextual challenges in EDs for each stage of the research process and thus facilitate patient engagement [15].

Respondents to the present survey expressed a positive view of patient engagement in the training of health students, which is validated by various studies showing a positive impact of patient engagement in education [22]. Unlike the mixed view of patients herein concerning the impact of patient engagement on training, other studies focusing on the experience of partner patients reported a positive view regarding educational experiences and the fact that patient partnership can highlight issues that would have otherwise been ignored [6]. Interestingly, although it is well known that healthcare safety is the basis of patient engagement, the respondents of the present survey did not raise improvement of safety as an advantage [12].

### Semi-structured interviews

The results obtained herein from the interviews of partner patients highlight the positive perception of these patients regarding patient partnership in the context of a service re-organization project. All reported being willing to continue working on the project. This is consistent with the expected benefits of patient involvement in service organization reported by the heads of EDs herein and with the literature describing positive experiences from patients and healthcare workers who expressed satisfaction with the engagement processes and reported being interested in continuing their involvement in the long term [6, 23].

However, a difference in perspective between partner patients and the heads of EDs concerning research and student training was observed. Indeed, while partner patient expressed the importance of their role in research less than a third of heads of EDs agreed to involve patients in the feasibility or implementation of research protocols. This difference could be explained by the particular challenges that EDs face in terms of research. As reported by Rising et al., patients treated in the EDs often lack a clear diagnosis and there is no clearly defined follow-up for many patients. Moreover, the diversity of patients and providers in the EDs pose challenges in terms of communication and delivery of high quality, empathic emergency care [24]. There is thus a necessity to adapt conceptual frameworks for patient engagement in EDs at the research level, as previously proposed [15], in order to improve outcomes for patients, families, and staff.

Although the perception of patient involvement in medical student training is more mixed among patients, the survey results highlight a positive impact and a formative experience of patient involvement according to a majority of the heads of EDs. Some studies have described patient engagement in the development of educational materials, tools, policy, and planning documents, including evaluation tools, to enhance care process or structural outcomes [6].

Patient partnership being a very time-consuming activity was an important drawback raised by the patients. The views of partner patients herein are in line with those reported in previous qualitative studies: improving patient-caregiver relationships in terms of communication, having a better understanding of the healthcare system, their wish to be involved in their direct healthcare, and the difficulties to accommodate their work and engagement [25, 26].

### Limitations of the study

The present study has several limitations. First, the low response rate to the survey may cause several selection biases, such as the selection of heads of EDs already interested in the patient partnership approach. There was also a higher response rate from teaching hospitals compared to non-teaching hospitals preventing a complete overview of patient engagement practices in EDs in France. Moreover the use of a questionnaire with mainly close-ended questions limits the information obtained and calls for a qualitative study to further explore these perceptions. The small sample size for the quantitative data analysis is also a major limitation as saturation was probably not achieved. In addition, the patients interviewed may have a different experience and opinion regarding patient involvement than those who participated in the actions reported by the heads of EDs (research and training) because they were involved in another level of the Montreal Model (design of healthcare services).

## Conclusion

This preliminary study highlighted a positive perception of patient engagement in EDs in France, from both the practitioner and patient perspective. Complementary studies are needed to assess the barriers and facilitators of patient partnership implementation, specifically in EDs in France, as this practice remains limited despite its known benefits.

### Electronic supplementary material

Below is the link to the electronic supplementary material.

Supplementary Material 1

## Data Availability

The datasets used and/or analyzed in the present study are available upon reasonable request to the corresponding author. Data transfer must be approved by the French data protection agency (Commission nationale de l’informatique et des libertés, CNIL).
