# Supplementary material for: Assessing patient partnership among emergency departments in France: a cross-sectional study
Source: BMC Health Serv Res. 2023 Aug 23;23:897. doi: 10.1186/s12913-023-09905-7 (PMC10463322; doi:10.1186/s12913-023-09905-7)
Supplement: Supplementary file 1 — Supplementary Material 1 [file 12913_2023_9905_MOESM1_ESM.docx]

**SUPPLEMENTAL MATERIAL**

**S1: Questionnaire**

***Respondent characteristics***

1. Gender:
2. Age:
3. Years of practice:
4. Type of hospital:
5. In which emergency department do you work?
6. Do you know the patient partnership approach?

We propose a definition: The patient partnership approach considers the patient or his caregiver as a partner of the healthcare team by considering the knowledge gained from life and disease (experiential) complementary to scientific or professional knowledge.

***Patient partnership in service organization***

1. Have you already involved patients in reorganization plans for your department in terms of functioning or premises?
2. If yes, indicate how

☐*Surveys conducted on patients*

☐*Personal interviews or focus groups*

☐*Consulting patient committees*

☐*Working groups involving patients and caregivers*

☐*Other*

1. *If yes, indicate for what type of project*

☐Service reorgazination

☐Developing care pathways

☐Installing signposting

☐Protocol development

☐Development of documents intended for patients

1. Do you think patient involvement could optimize patient pathway?

☐1 Strongly agree

☐

☐

☐

☐5 Strongly disagree

1. Do you think patient involvement could improve the layout of premises?

☐1 Strongly agree

☐

☐

☐

☐5 Strongly disagree

1. Do you think patient involvement could improve patient experience regarding care?

☐1 Strongly agree

☐

☐

☐

☐5 Strongly disagree

***Patient partnership in research***

1. Do you have research protocols in your service?
2. Have you already involved patients in the feasibility or implementation of research protocols?
3. If yes, precise how

☐ Surveys conducted on patients

☐ Personal interviews or focus groups

☐ Consulting patient committees

- Working groups involving patients and caregivers

1. If yes, precise at which stage

☐ Pilot committee of the study

☐ Protocol development

☐ Ethical considerations

☐ Patient enrollment

☐ Result reporting

1. If yes, precise the type of patients involved

☐ Individual patients

☐ Patient representatives

- Patient relatives

1. Do you think that patient involvement is important for the feasibility or implementation of research protocols?

☐1 Strongly agree

☐

☐

☐

☐5 Strongly disagree

***Patient partnership in training and education***

1. Have you already involved patients in student training and education?
2. For which student division do you think patient involvement plays a positive role?

☐ Nurse

☐ Medical student and medical resident

☐ Caregiver assistant

1. Do you think that patient perspective is important concerning the role of student in emergency departments?

☐1 Strongly agree

☐

☐

☐

☐5 Strongly disagree

1. Do you think that patient experience is formative for health students in emergency departments?

☐1 Strongly agree

☐

☐

☐

☐5 Strongly disagree

***General considerations in emergency departments***

1. What would be the benefits of patient partnership in emergency departments? Give 3 benefits ranked from the most important to the least important
2. What would be the downsides of patient partnership in emergency departments? Give 3 downsides ranked from the most important to the least important
3. Would you be interested in integrating patients as partners in

☐Service organization

☐Research projects

☐Teaching and education

1. Comments:

**S2: Patient interview guide**

**Introduction**

1) Could you introduce yourself? What is your experience as a patient? What is your experience with our healthcare system?

2) Why did you become a partner patient? How did you join Bauréals project (design of a future building in a French teaching hospital)? What were your motivations? Did you have a prior training?

3) What was your work for Bauréals? How were the workshops animated? Which method was used for your participation? How much time did it represent? Did you receive a financial compensation? Did the workshops correspond to what you expected?

4) Were the contributions expected from you clearly explained?

5) On which aspects did they ask your perspective? Are there other aspects where patient perspective is important?

6) How did you perceive the welcome you received from other participants in the workshops? Can you tell me more about your relationship with them?

7) Did you recognize the different categories of participants of the workshops and their missions?

8) How do you consider your impact on the project? Did you feel that your work was valued? Did you feel legitimate in participating? Why?

9) Did you feel capable enough? If not why?

10) Do you want to continue to be a partner patient? What would push you to continue? What would hold you back? How do you see your implication in the future?

11) Comments?
